# Adjustable Fluorescence Emission of J-Aggregated Tricarbocyanine in the Near-Infrared-II Region

**DOI:** 10.1021/acs.jpcb.3c04554

**Published:** 2023-09-08

**Authors:** Nitzan Dar, Haim Weissman, Rinat Ankri

**Affiliations:** † Department of Physics, Faculty of Natural Science, Ariel University, Ariel 40700, Israel; ‡ Department of Molecular Chemistry and Material Science, The Weizmann Institute of Science, Rehovot 7610001, Israel

## Abstract

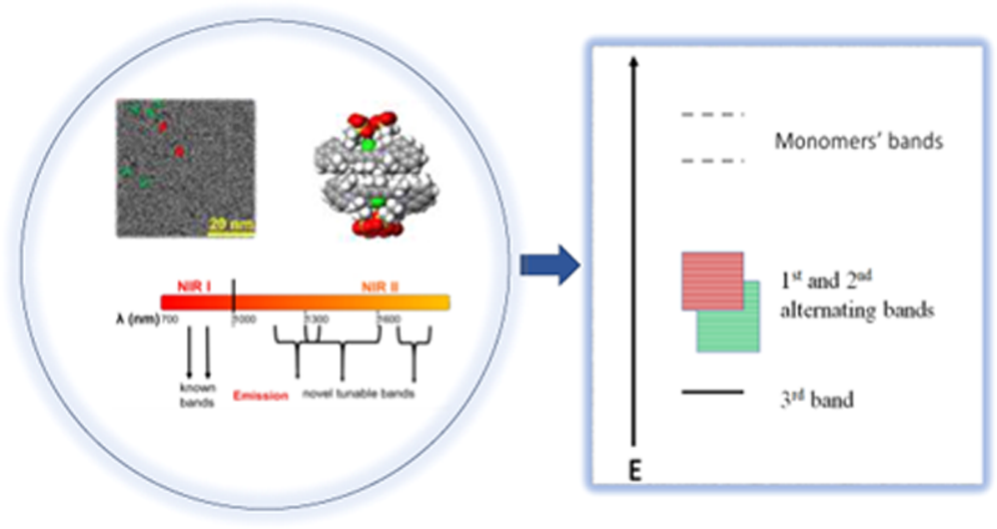

Near-infrared (NIR) J-aggregates attract increasing attention in many areas, especially in biomedical applications, as they combine the advantages of NIR spectroscopy with the unique J-aggregation properties of organic dyes. They enhance light absorption and have been used as effective biological imaging and therapeutic agents to achieve high-resolution imaging or effective phototherapy in vivo. In this work, we present novel J-aggregates composed of the well-known cyanine molecules. Cyanines are one of the few types of molecules whose absorption and emission can be shifted over a broad spectral range, from the ultraviolet (UV) to the NIR regime. They can easily transform into J-aggregates with narrow absorption and emission peaks, which is accompanied by a red shift in their spectra. In this work, we show, for the first time, that the tricarbocyanine dye (IR 820) has two sharp fluorescence emission bands in the NIR-II region with high photostability. These emission bands can be tuned to a desired wavelength in the range of 1150–1560 and 1675 nm, with a linear dependence on the excitation wavelength. Cryogenic transmission electron microscopy (cryo-TEM) images are presented, and combined with molecular modeling analysis, they confirm IR 820 π-stacked self-assembled fibrous structures.

## Introduction

Fluorescence-based nonradiative bioimaging in the range of 1000–1700 nm (known as the second near-infrared window, NIR-II) has gained great attention owing to its high spatiotemporal resolution and deep penetration depth.^1,2^ The optical bioimaging in this spectral window shows minimal photon scattering and almost zero tissue autofluorescence, providing high spatial resolution and a high signal-to-noise ratio.^1,3^ As a result, organic NIR-II emitters have attracted widespread attention due to their own advantages, including relatively smaller molecular weight, easier functionalization, shorter retention time in organisms, and more.^4^

However, it is difficult to extend both the maximum absorption and emission wavelengths beyond 1300 nm of organic emitters through structural modification. J-aggregates, the highly ordered arrangement of organic dyes,^5^ offer an excellent way to bathochromic-shift the absorption and emission wavelengths of the organic emitters to the NIR-II window.^5,6^ These aggregates often show high functions, originating from their structures, which are quite different from those of isolated molecules or crystals. Previous reports on J-aggregates mainly focused on the assembly of organic dyes to improve their stability in physiological environments, such as in DNA^2^ or lipid bilayers,^7^ and polymers.^8^ However, the formation of these supramolecular architectures by self-assembly, which obtain a well-defined microscopic organization and macroscopic characteristics, is still a significant chemical challenge.

Herein, we report novel J-aggregates, with tunable fluorescence properties in the NIR-II regime, varying between 1100 and 1700 nm, using the tricarbocyanine (IR 820) biocompatible cyanine dye. Cyanine dyes are a fascinating topic for both basic and applied science. Their structure is based on a polymethine chain of various lengths. These chains contain a single positively charged nitrogen atom, usually as part of a heterocycle, which is surrounded by a counteranion.^5,6,9^ Consequently, these molecules have a high absorption coefficient and high fluorescence emission intensity. Cyanines’ absorbance and emission can be shifted in a broad spectral range from the ultraviolet to the NIR,^10,11^ depending on the polymethine chain length and its terminal substituents.^12^ They own tendency to self-assemble into different topologies including dimers, single- and double-walled nanotubes, bundles, and sheets^6^ in a form of J-aggregates, which results in much narrower absorption and emission peaks, along with a red shift in their spectra compared to cyanine dye monomers. Due to their unique spectroscopic properties, cyanine dyes have numerous applications, such as in solar cells, photovoltaic layers,^13^ light harvesting,^14^ chemical sensors,^15,16^ shortwave infrared emitters,^17^ and in vivo imaging.^17−19^ Thus, e.g., a few tricarbocyanine (heptamethine) dyes, such as indocyanine green (ICG), which emits at 830 nm and is in wide use in clinical applications, and the IR dye 800CW, which emits at 810 nm, were utilized in preclinical studies.^20^

In this work, we investigate the spectral properties of the commercially available IR 820 and its tendency to form J-aggregates, to produce an NIR-II emission. Previous studies on IR 820 have presented fluorescence emission in the NIR-I regime. Thus, e.g., Yen et al. have shown IR 820 reacted with ethylenediamine conjugated onto poly(isobutylene-alt-maleic anhydride) and grafted on a ferric oxide nanoparticle (NP), while each part of the multistep process led to a spectral shift in the emission peak, from 1064 nm for bare IR 820 dyes to 864 nm in the composition with a polymer and ferric oxide NP.^21^ Feng et al. presented IR 820 emission peaks at 829 nm (water) and 858 nm (fetal bovine serum), used for the imaging of the middle cerebral artery occlusion in a mouse model.^22^ In another study, the IR 820 was reacted with dicarboxyphenyl, encapsulated into liposomes, and was then utilized for mice spleen imaging at 934 nm.^23^ In another research, a sulfonated IR 820 complexed with the protein human serum albumin for bioimaging has shown a tail fluorescence emission at 1000–1150 nm, and the full structure of the peak/s was not shown.^24^ Absorbance and emission spectra of IR 820 in MeOH and water at different concentrations were also investigated by Fernandez et al.,^25^ and they showed a large solvatochromism effect in absorbance of IR 820.^26^ The emission peak at 822 nm was independent of concentration and also of the excitation wavelength which was either 691 or 785 nm. Despite the intensive work done on the spectral properties of IR 820, previous studies have focused on its emission in the NIR-I region, while none of them have shown emission peaks in the NIR-II region (<1100 nm, ≥272.5 THz).

In this work, we present the tunable fluorescence of J-aggregated IR 820 dye in the near NIR-II region. Fluorescence within the NIR-II region was captured, revealing a novel observation of two distinct and pronounced J-emission bands. These bands exhibit a considerable Stokes shift (400–1000 nm) in comparison to the absorbance spectra, as seen in various aqueous solutions of IR 820. This finding represents a significant advancement in our understanding of NIR-II fluorescence behavior. We show that the dye has two emission bands that can be tuned over the range of 1150–1560 nm and 1675 nm, with a linear dependence on the excitation wavelength. Complementary studies on the photostability of these solutions, nuclear magnetic resonance (NMR), dynamic light scattering (DLS), and fluorescence lifetimes were performed. Cryogenic transmission electron microscopy (Cryo-TEM) was performed to analyze the morphology of the aggregates, and with appropriate modeling of the aggregated molecules, we prove the IR 820 J-aggregated structure.

## Methods

### IR 820 Preparation

IR 820 was purchased from Angene (London, England) and dissolved in ethanol AR (Bio-Lab, Israel) to form a 0.253 mM ethanolic stock solution. For each experiment, the ethanolic solution was diluted in one of the following solvents: double distilled (dd) water, sodium chloride (Chem-Lab, Belgium) aqueous solutions, sodium sulfate and sodium hydroxide (Chem-Lab, Belgium), phosphate-buffered saline (PBS, purchased from Biological industries, Israel), chloroform (AR, purchased from Bio-Lab, Israel), or dimethyl sulfoxide (DMSO, baker analyzed, purchased from J.T Baker, Poland).

### Absorption, Emission, and Fluorescence Lifetime Measurements

Absorbance spectra were measured with an FP-8500 spectrofluorometer (Jasco, Japan), and the fluorescence emission, fluorescence lifetime (FLT), and photostability spectra were measured using a Fluorolog-Quanta Master (Horiba scientific, Japan). Data was analyzed using FelixFL software (version 1.0.33.0, Horiba scientific, Japan). 1 mm optical path length quartz cuvettes were used for both absorbance and fluorescence measurements. Fluorescence lifetime measurements were performed using the time-correlated single-photon counting (TCSPC) method, with a thermally cooled Hamamatsu NIR PMT photon detector. The lifetime measurements were carried out using a delta diode of 830 ± 10 nm, with a peak wavelength at 819 ± 10 nm, an extremely narrow 50 ps pulse width, a 0.6 mW average power, and a 100 MHz repetition rate. The fluorescence decay curves were analyzed using FelixFL decay analysis software based on a multiexponential model which involves an iterative reconvolution process.

### Nuclear Magnetic Resonance (NMR) Measurements

^1^H NMR of 1.2 mg of IR 820 dissolved in 2 mL of DMSO-d6 (Andover, MA) was recorded using a 400 MHz NMR spectrometer (Bruker BioSpin, Rheinstetten, Germany). Chemical shifts were reported in parts per million (ppm) units, relative to tetramethyl silane (TMS) as an internal standard.

### Cryogenic Transmission Electron Microscopy (cryo-TEM)

Vitrified samples were prepared by applying 6.5 μL of each sample to a 200 mesh copper grid coated with holey carbon (Pacific Grid-Tech supplies). The samples were blotted at 23 °C and 95% relative humidity and plunged into liquid ethane using a Leica EM-GP automatic grid plunger. Specimens were introduced into a microscope using a Gatan 626 cooling holder and transfer station and were equilibrated at −178 °C in the microscope prior to the imaging.

Imaging was performed using a Tecnai G2 TWIN-F20 microscope equipped with a field emission gun (FEG) operating at 200 kV. Images were recorded using a TVIPS TemCam-XF416(ES) CMOS digital camera or a Tecnai T12 transmission electron microscope operated at 120 kV equipped with a TVIPS TemCam-XF416 CMOS digital camera.

### Dynamic Light Scattering (DLS)

Size distribution measurements of the aggregates were carried out using the dynamic light scattering (DLS) system LitesizerTM 500 Particle Analyzer (Anton Paar, AUT) at ambient temperature. Calculations were done using the refractive index of water (*n* = 1.33).

## Results

### Absorbance and Emission of IR 820 in Aqueous Solutions

**Figure 1:**
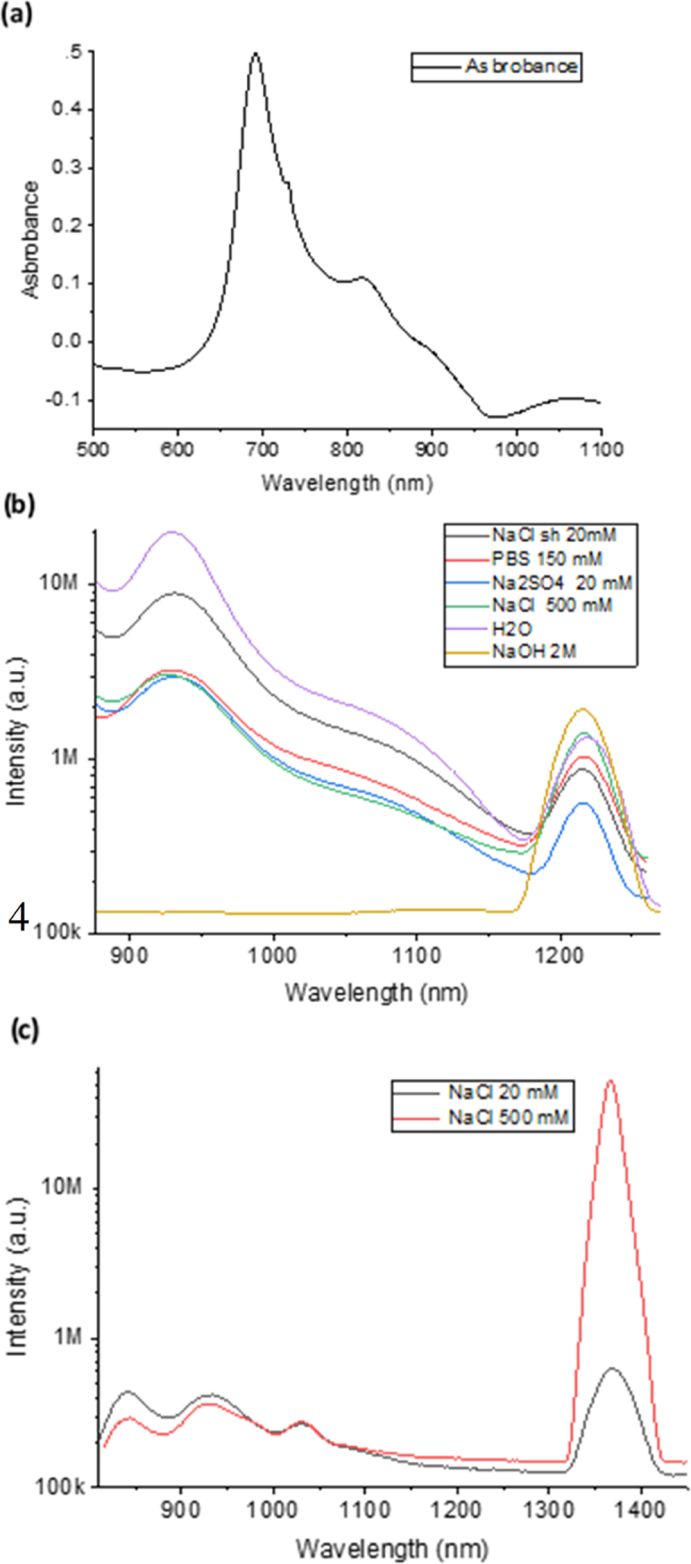
Absorption and emission spectra of IR 820 in different aqueous solutions. (a) Absorption spectrum of 23 μM IR 820 in water. (b) IR 820 emission spectra in various concentrations of H2O, NaOH, NaCl, Na_2_SO_4_, and PBS and excitation wavelength: 810 nm. (c) Emission spectra of IR 820 dissolved in 20 and 500 mM NaCl aqueous solutions, following 685 nm excitation wavelength. The scale is logarithmic.

Due to the amphiphilic properties of IR 820 and its aggregation tendency,^22,25^ we studied its fluorescence photophysical properties in various aqueous solutions, including the biocompatible PBS, and in a wide spectral range of 815–1770 nm. IR 820 has a relatively high molar absorption coefficient of 7.2 × 10^4^ M^–1^ cm^–1^;^22,27^ therefore, its measurements were performed at a relatively low concentration of 10^–5^ M. Analysis of the absorption peaks resulted with the most prominent peaks at ∼690 and ∼815 nm (Figure 1a), similar to previously reported results.^25^ The emission fluorescence spectra of IR 820 in the following aqueous solutions, H_2_O, NaOH, NaCl, Na_2_SO_4_, and PBS, are presented in Figure 1b, showing an emission peak at 930 nm and an additional prominent sharp peak at 1217 nm (FWHM of 25–40 nm), providing a very large Stokes shift of ∼400 nm. One can observe that the 1217 nm peak intensity, position, and width are independent of the solute, corresponding with J-aggregate nature.^6,28^ Even though the ratio between the two emission peaks varied, the higher the salt concentration, the higher the 1217 nm emission peak. The emission from IR 820 when dissolved in NaOH was remarkably intense, leading to the subdued visibility of the monomer’s peak. The predominant observation of IR 820 dye in the form of J-aggregates further accentuates this phenomenon (the light brown line in Figure 1b).

As was previously reported, the excitation wavelength might change the emission peak of different dyes;^25^ therefore, we have investigated the dependence of IR 820 emission on the excitation wavelength. IR 820 dissolved in NaCl aqueous solutions at concentrations of 20 and 500 mM were excited at 685 nm (Figure 1c). One can observe a J-band emission peak that was shifted from 1217 to 1365 nm, while a 930 nm peak remained at the same position. At 500 mM concentration of NaCl, the J-band emission peak was 2 orders of magnitude higher compared to the 20 mM NaCl solution (Figure 1c).

**Figure 2:**
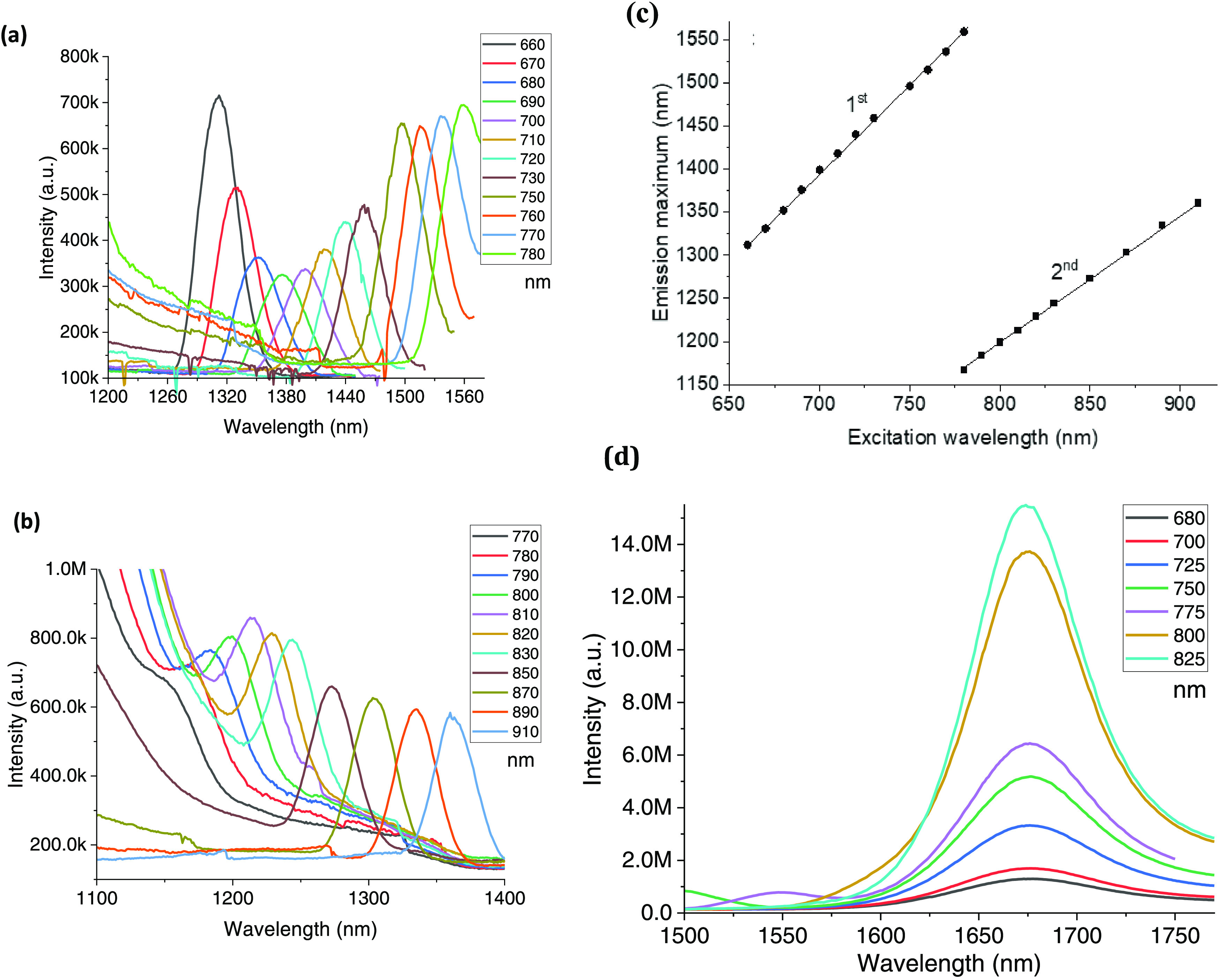
Emission spectra of IR 820 at excitation wavelengths of 660–910 nm in water. (a) 1st emission band in the range of 660–780 nm. (b) 2nd emission band in the range of 770–910 nm. (c) Dependencies of the 1st and 2nd emission peak on excitation wavelengths, each circle (1st band) or square (2nd band) represents an experiment. (d) 3rd emission band in the range of 680–825 nm.

**Figure 3:**
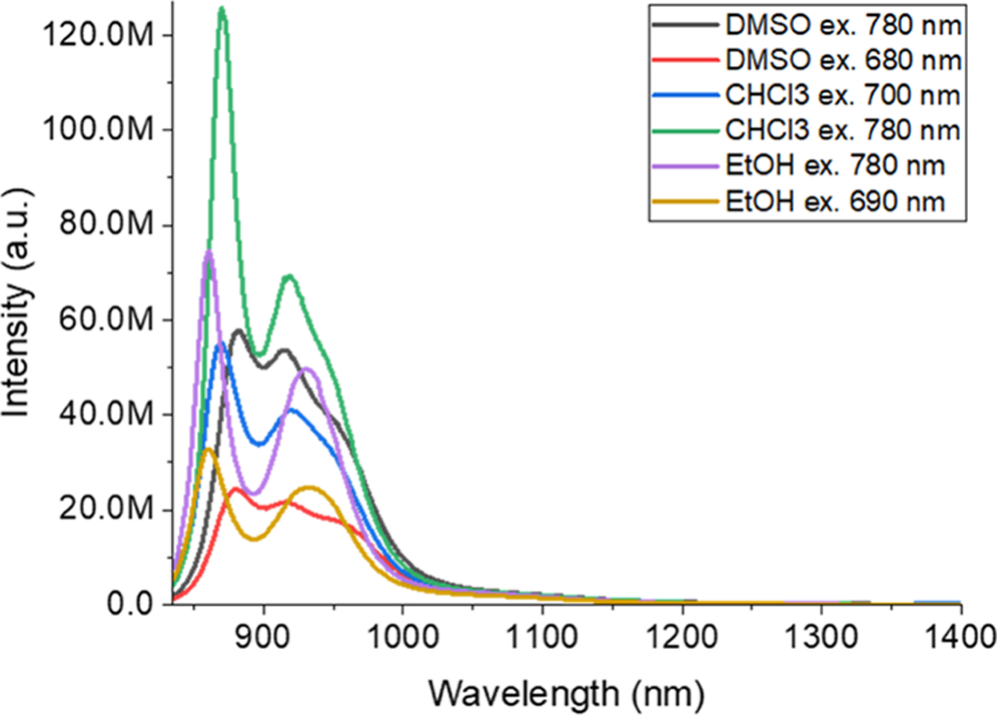
Emission of IR 820 in ethanol, chloroform, and DMSO at two different excitation wavelengths (nm).

Following these promising results, which shows a spectral dependence of IR 820 on the excitation wavelength, we then studied the relationship between the position of IR 820 J-band and the excitation wavelength (Figure 2). The excitation wavelengths varied between 660 and 910 nm to cover most of its absorption range. We discovered three different emission bands, while each band has a different spectroscopic characterization. Figure 2a shows a series of different excitation wavelengths, in the range of 660–780 nm, defined as the 1st emission band, while increasing the excitation wavelength caused a red shift of the emission band. Interestingly, when the excitation was larger than 780 nm, the emission band disappeared, probably due to forbidden transitions in these wavelengths. To ensure that a second-order diffraction is not affecting the emission results, we used a short pass 800 nm 2″ filter (SPF-800-2.0, CVI Laser Optics) in three representative excitation wavelengths. The emission spectra following these representative experiments showed a similar pattern to results presented in Figure 2a (see Figure S5, in the Supporting Information), suggesting that the fluorescence is not due to second-order diffraction. Figure 2a shows a linear dependence between the emission wavelength and the excitation. Figure 2b shows another emission band (2nd emission band), following a series of different excitation wavelengths, in the range of 770–910 nm. When the 1st emission band disappears, the 2nd band emerges, with a pattern similar to the 1st emission band: increasing the excitation wavelengths resulted with a red shift of the emission band, with a linear relationship between the excitation wavelengths and the emission peaks (Figure 2c). Examination of Figure 2c suggests that by fine-tuning the excitation wavelength, one can achieve a specific desired emission band. In addition, results show that when the transitions are allowed for the 1st band, 2nd transition band is forbidden and vice versa. The transition excitation wavelength between these alternating bands is around 780 nm (Figure 2a,b). On the contrary, the 3rd emission band emits at a constant wavelength of 1675 nm for a wide range of excitation wavelengths Figure 3d. Interpreting the results suggest that IR 820 has two energy levels in the NIR-II region, while one of them can be tuned according to the excitation wavelength and is alternating to a different emission range. Conversely, the other energy level remained at a constant energy in the excitation range.

Noteworthily, when we tried to see the emission peaks of the IR 820 dye dissolved in organic solvents, rather than aqueous solution, we did not find any peaks between 1000 and 1400 nm (see Figure 3). Since these sharp emission peaks appeared only in aqueous solution, we conclude that aqueous conditions are required for the J-aggregate formation.

### Dependence of the IR 820 Emission Peak on Solute Concentration

**Figure 4:**
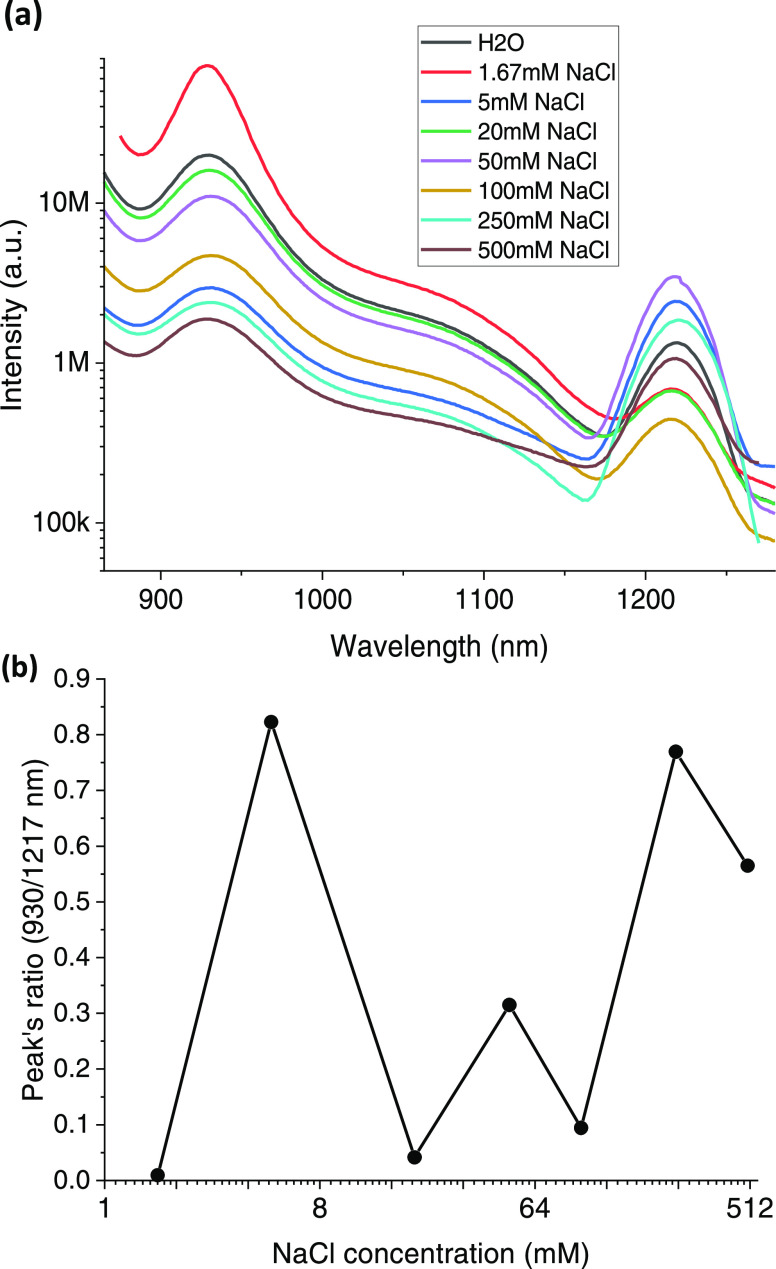
(a) Emission spectra of IR 820 at different concentrations of NaCl aqueous solutions at an excitation wavelength of 810 nm. (b) Correlation between the fluorescence intensities, calculated from the ratio between the peak intensity at 930 and 1217 nm.

To find the dependence of the IR 820 J-band on the solute concentration, we performed a series of consecutive emission measurements following a 810 nm excitation, in which we changed the concentration of NaCl while keeping the concentration of IR 820 constant (Figure 4). In contrast with former results, which showed a large increase in the J-band in comparison to the 930 nm monomer’s band (Figure 1c), we found that the ratio between the two emission peaks, at 930 and 1218 nm, changed in a much more complexed manner. For example, 5 mM NaCl has the most prominent J-band, while 100 mM NaCl has the weakest J-band (Figure 4b).

### cryo-TEM and DLS of IR 820 in Water

**Figure 5:**
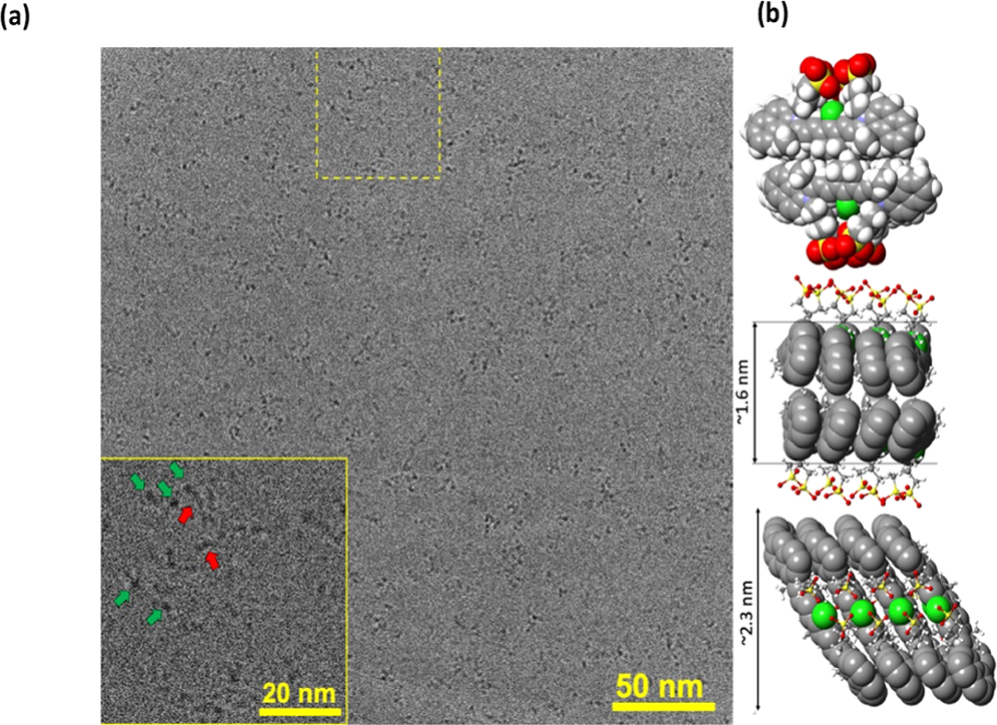
Dimensions and morphology of the IR 820 in water. (a) cryo-TEM micrograph of IR 820 fibers. (b) The molecular model of the IR 820 dimer was partially energy-minimized to fit the observed dimensions.

cryo-TEM imaging IR 820 dye, an aqueous solution revealed the formation of the self-assembled fibers. The length of the observed fiber in the micrograph below was found to be as long as 9.5 nm, with an average diameter of 1.6 nm (Figure 5a). The observed short fibers and the high contrast of their cross sections, typical to an extended stacked π systems,^29,30^ may be explained by the formation of short bilayered J-aggregated fibers based on the presented molecular modeling (Figure 5b). The molecular modeling utilized sequential partial energy minimizations utilizing MM2 and MM3 force fields. The modeling indicates a possible but unfavorable π–π aggregation due to steric interactions of the bulky methyl moieties where a bilayer character is needed to stabilize the hydrophobic side of the short molecular column.

In Table S1, in the Supporting Information, we show the calculations of the different cross sections of the aggregation structures presented in Figure 5b. The cross sections have statistical variance between its width (1.6 ± 0.1 nm, averaged over 719 lines) and length (2.1 ± 0.2 nm, averaged over 908 lines), which corroborates the suggested values in the molecular model. The average of the measured values (1.7 ± 0.1 nm, averaged over 652 lines) is in statistical agreement with the value of the narrower side of the cross section. This is expected as for the fibers, there will be a strong bias toward due to the expected differences in thickness. The wider faces of the fibers will have an average thickness of 1.6 vs 2.1 nm, thus making their observation in the micrograph less probable.

DLS showed that the hydrodynamic diameter of IR 820 in water is 3.35 ± 1.5 nm (Figure S4, Supporting Information). The difference in size obtained by the Cryo-TEM and the DLS can be explained through the fact that in DLS, the aggregates are considered as spheres, in contrast to their real fibrous structure, as well as the fact that the values obtained by DLS measurements represent the hydrodynamic diameter of a sphere (i.e., the diameter of a particle with a hydration shell).

### Photostability and Fluorescence Lifetime of IR 820 in Water

**Figure 6:**
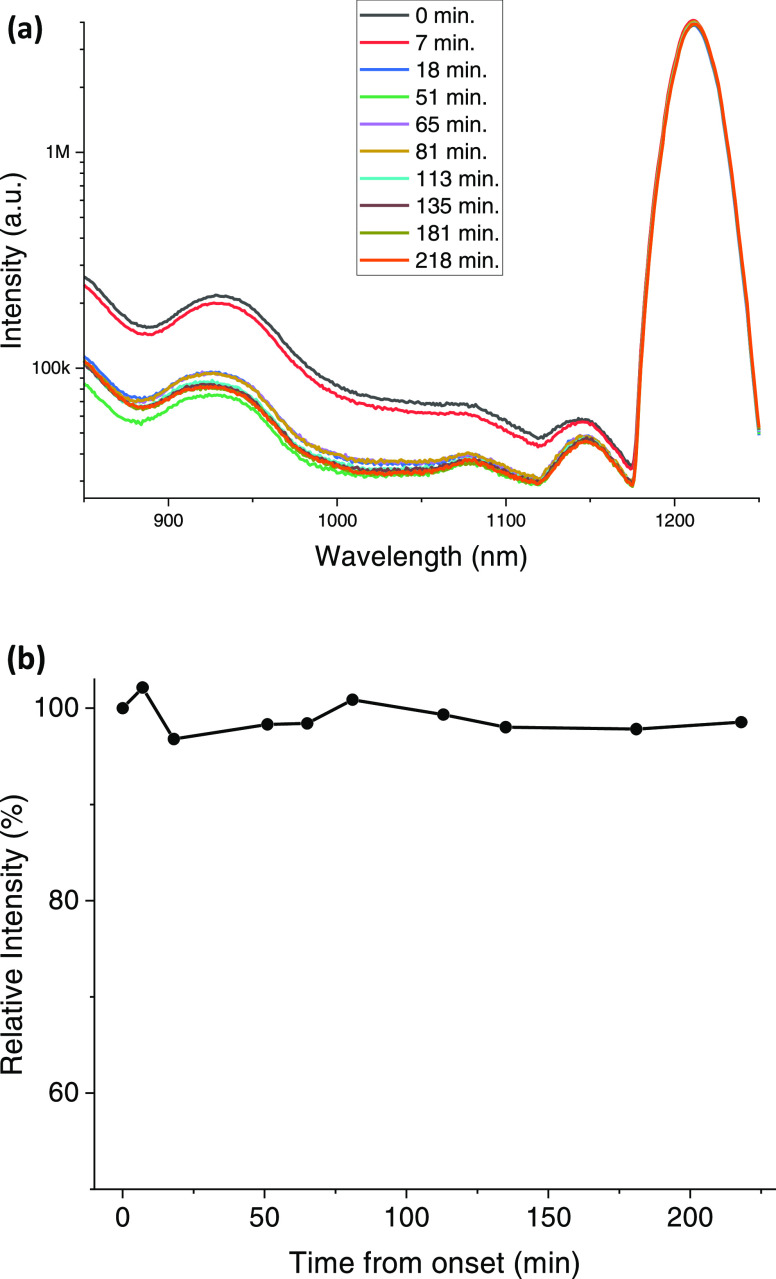
Consecutive emission spectra of IR 820. (a) Fluorescence intensity of IR 820 versus time. Each colored line indicates the time of measurement from onset. (b) 1212 nm peak relative fluorescence intensity versus time. Each dot is a recorded fluorescence measurement (at time *t* = 0, the intensity was 100%).

As discussed in the Introduction section, highly stabilized J-aggregates are difficult to synthesize. In this section, we show that our J-aggregated IR 820 molecules show a relatively high stability in time. IR 820 photostability experiments were conducted through experiments in which we recorded the emission spectrum of IR 820 every 20–30 min, for 10 consecutive measurements (Figure 6a,b). Analysis of the relative intensity of the 1212 nm emission peak to the 1st measurement during each measurement, when the 1st emission at the onset was defined as 100%, is shown in Figure 6b. Results show that the IR 820 prominent 1212 nm peak remains stable for at least 218 min, which agrees with a recent reported study,^31^ but it contrasts with its reported instability under laser irradiation.^22,32^ One can also observe that the emission intensity at 924 and 1150 nm decreased after 23 min, while the 1212 nm aggregate peak remains stable.

The fluorescence lifetime of IR 820 dissolved in water was measured using our TCSPC setup, as described in the Methods section. The observed FLT was 89 ± 2 and 16 ± 1 ps in 20 and 500 mM NaCl solutions, respectively. These values are similar to the ones reported by Berezin et al.,^26^ while a most possible reason for the short lifetime is the superradiance of the J-aggregates.^33^

### NMR measurements of IR 820

^1^H NMR spectra of IR 820 dissolved in D_2_O are presented in Table 1. Shifts of the cyanine chain and the aromatic rings were observed, which are similar to previous reported results.^34^ The NMR spectral graph is shown in the Supporting Information (Figure S6). Results suggest that the IR 820 was not degraded or mixed with other impurities.

**Table 1:** Chemical Shift Report of IR 820 ^1^H NMR

^1^H NMR (400 MHz, DMSO)
δ 8.41–8.34 (m, 1H), 8.33–8.27 (m, 1H), 8.11–8.04 (m, 2H), 7.86–7.78 (m, 1H), 7.70–7.61 (m, 1H), 7.57–7.47 (m, 1H), 6.48–6.37 (m, 1H), 4.42–4.28 (m, 2H), 2.84–2.72 (m, 1H), 2.04–1.71 (m, 12H)

## Discussion

In this work, we present, for the first time, a unique behavior of the NIR-I dye, IR 820, with a tunable emission in the far-NIR-II regime, in the range of 1150–1725 nm. While the 830 and 929 nm peaks in the NIR-I region are attributable to the monomeric form of the dye, the NIR-II peaks, which are attributable to the J-aggregated structure of this molecule, can be tuned to a broad range of wavelengths up to ∼1725 nm. These bands are sharper than the bands of the monomer and are not prone to solvatochromism. They appear only in aqueous solutions and indicate the formation of J-aggregates.

Few NIR-II dyes have been reported to present a respectively large Stokes shift (∼400 to ∼600 nm), such as diketopyrrolopyrrole, fluorothiophene copolymer, or FD-1080 cyanine.^35,36^ FD-1080 is a cyanine molecule, with a structure similar to the IR 820 dye, and thus, its J-aggregation mechanism and Stokes shift might present a similar behavior. The other dyes might show another mechanism for Stokes shifting. However, none of those dyes have a large Stokes shift up to 1000 nm, as have been shown in this paper (Figure 6). A few theoretical explanations for the high red shift have been suggested, such as increasing the conjugation length separating the electron donor/acceptor and heteroatom substitutions.^27−38^ Still, since such large Stokes shifts and sharp absorption are barely found, those sharp, highly shifted peaks may come from a different source.

**Figure 7:**
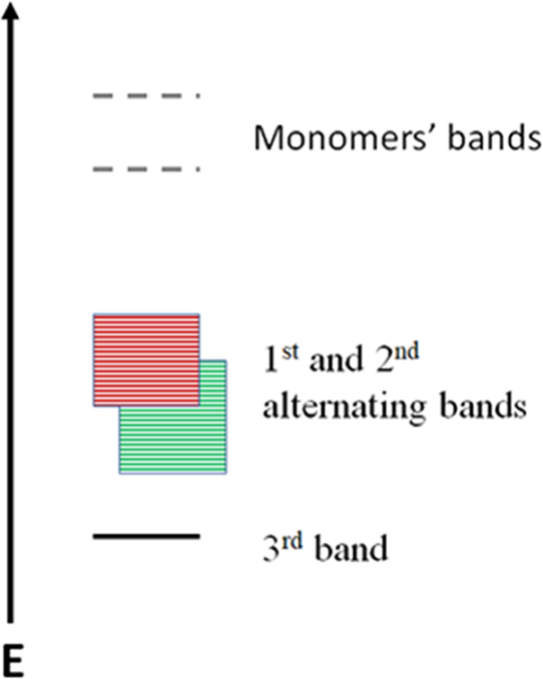
Scheme of IR 820 energy levels (not to scale).

The dependence of the IR 820 emission and its complexes on the excitation wavelengths has been previously reported.^25,39^ However, none of these works have reported the emission of IR 820 in NIR-II. Therefore, it is difficult to deduce an explanation for the alternating emission in the NIR-II regime, from those studies. In Figure 7, we suggest a new theory, with a new energy level diagram of IR 820, according to our results presented in Figure 2. The two monomers possess the highest energy levels. The 1st and the 2nd bands have an alternating pattern and can be tuned according to the excitation energy, while the lowest, 3rd energy level is constant. Several former studies have analyzed the energy level of dyes, suggesting few theoretical calculations, such as density functional theory (DFT) or isomerization and singlet triplet intersystem crossing.^40−42^ However, they do not show any tunable emission band, as our results show.

Our cryo-TEM micrographs and molecular modeling indicated a possible but an unfavorable π–π aggregation due to steric interactions of the bulky methyl moieties with a bilayer character (Figure 5b).^43^ The other evidence for the presence of the J-aggregates is the superradiance behavior of the dyes, presented by its high fluorescence, measured short FLT in the range of picoseconds, and the bathochromic shift.^5^ The J-aggregate formation is also in accordance with the excitonic theories^44,45^ and is also presented in other amphiphilic cyanine dyes.^46−49^ Our results also suggest that the solubility of the IR 820 affects the formation of its J-aggregates and enabled this formation in aqueous solutions only.

The important advantage of these amphiphilic cyanine dyes is that their self-assembly in aqueous solution can be controlled by the relative size of the hydrophobic side chains and the polar head groups. Accordingly, highly ordered J-aggregates of various morphologies became accessible from the same chromophore, and their characteristic optical properties could be related to the mutual orientations of the transition dipole moments of the dyes in the supramolecular arrangements.^9^ In Figure 4, we show that the relationship between the concentration of NaCl and the ratio between the 929/1217 emission peaks is quite complex and does not present a consistent behavior. A former study, which has presented a dependence of the emission intensities on the excitation wavelength has explained this behavior by the influence of the molecular orbital structure of the excited state, as well as the solvation shell.^50^ In another study, in which two tricarbocyanines were excited in alcoholic solvents, such as methanol, ethanol, propanol and butanol, they show a similar behavior, in which the emission peaks’ ratio depends on the polarity of the solvent.^51^ Yet, in both cases, the authors have investigated the monomer’s peaks rather than the aggregated form such as in our case, suggesting an ion pair formation for the most polar alcohols.

## Conclusions

The present work shows the tunable fluorescence emission of the J-aggregated cyanine dye IR 820 in the NIR-II region. The emission bands can be set in a range of ∼1150 to ∼1560 nm and 1675 nm, depending on the excitation wavelength. Addition of NaCl to the water solution has shown that the relationship between the polarity of the solvent and the emission peak of the J-band is quite complex. The morphological cryo-TEM image confirms the presence of aggregates in the form of π-stacked self-assembled fibers, and complementary NMR and DLS measurements also demonstrate the presence of IR 820 as an aggregated form. The photostability of the IR 820 dye in water was observed for more than 3.5 h, indicating that the dye can be used for a relatively long time without degradation. We show that the tricarbocyanines dye IR 820 has a much broader emission bandwidth than was previously shown and can therefore be used for many applications, such as chemical sensing and biomedical imaging, making it a bright, biosafe organic chemical for in vivo imaging.

## Supporting Information Available

Cryo-TEM images in additional resolution widths; molecular cross section statistics of the aggregated IR 820; DLS size distribution of IR 820; fluorescence spectra of IR 820 using a 800 nm short pass filter; and ^1^H NMR spectra of IR 820
(PDF)
