# Supplementary material for: Adjustable Fluorescence Emission of J-Aggregated Tricarbocyanine in the Near-Infrared-II Region
Source: J Phys Chem B. 2023 Sep 8;127(37):7988–95. doi: 10.1021/acs.jpcb.3c04554 (PMC10518818; doi:10.1021/acs.jpcb.3c04554)

Supporting information; Adjustable Fluorescence Emission of J-aggregated Tricarbocyanine in the Near Infrared-II Region

Nitzan Dar[1*], Haim Weissman[2] and Rinat Ankri[1*]

1 Department of Physics, Faculty of Natural Science, Ariel University, Ariel 40700, Israel

2 Department of Molecular Chemistry and Material Science, The Weizmann Institute of Science, Rehovot, 7610001, Israel

*Corresponding authors: rinatsel@ariel.ac.il

Content:

Figures S1-S3: Cryo TEM images in additional widths

Table S1: Molecular cross sections of the aggregated IR 820

Figure S4: DLS size distribution of IR 820

Figure S5: Fluorescence spectra of IR 820 with 800< nm short pass filter

Figure S6: ^{1}H NMR spectra of IR 820

Figure S1: Measurements of widths 1

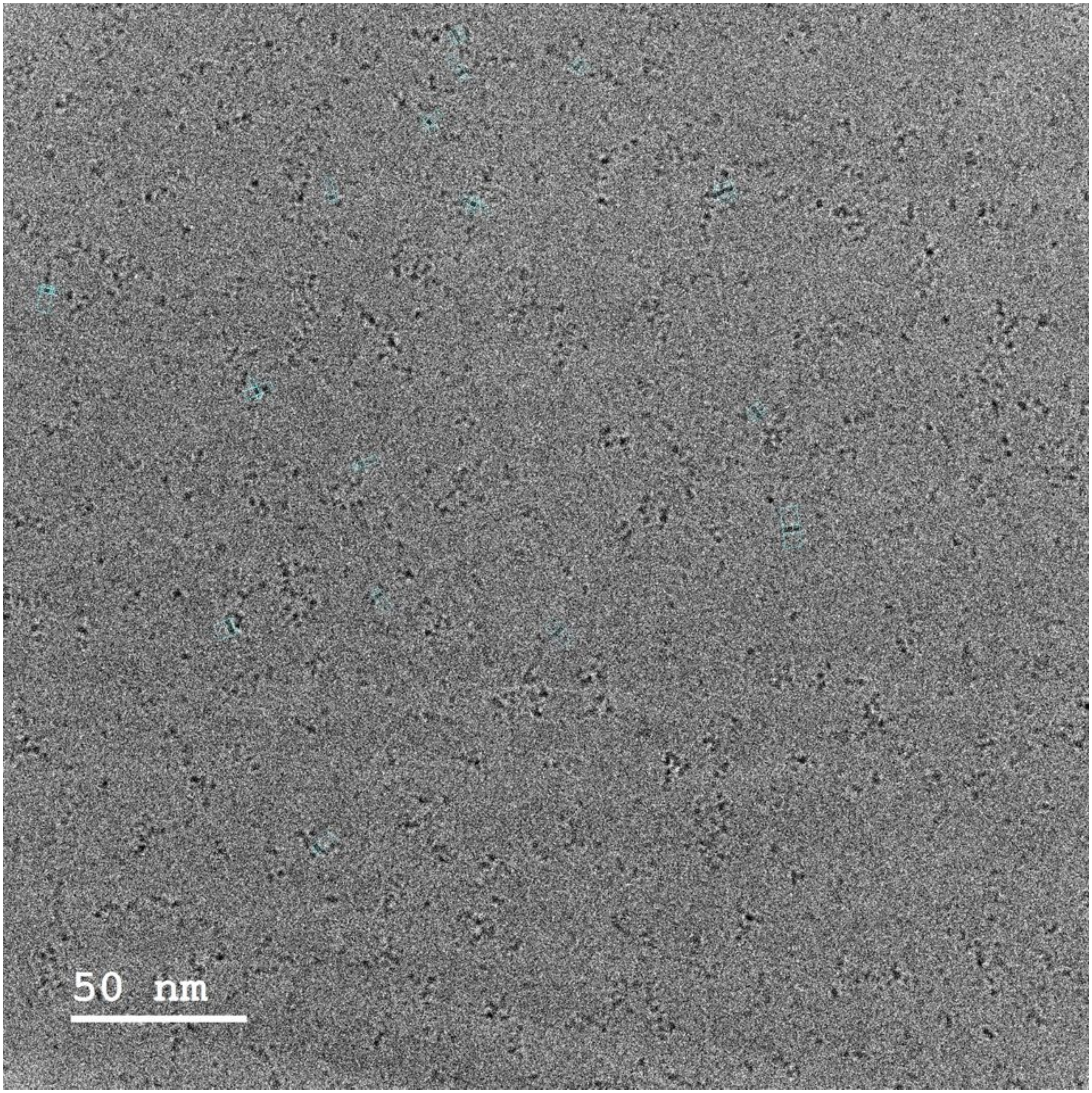

Figure S2: Measurements of widths 2

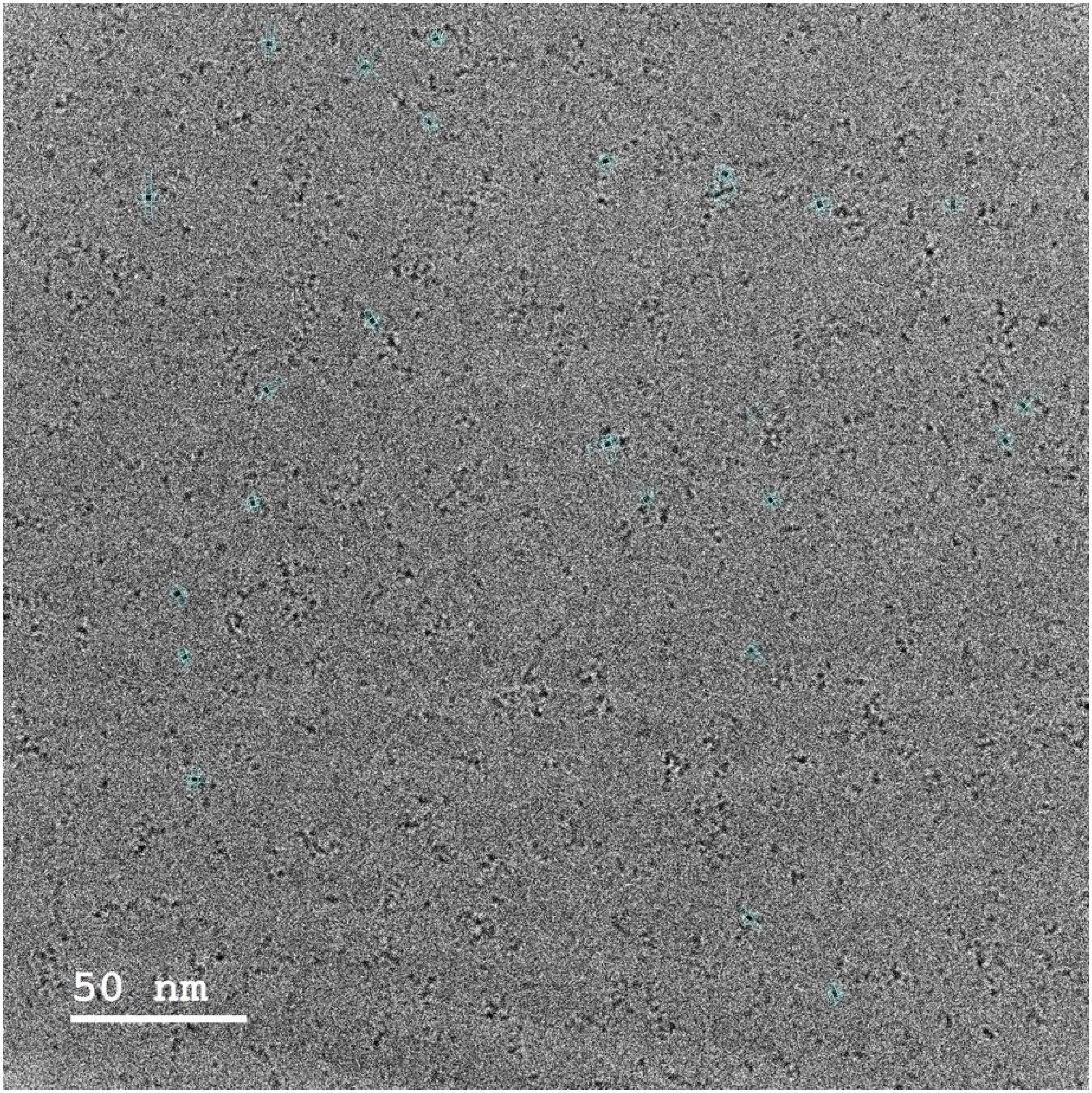

Figure S3: Measurements of lengths 2

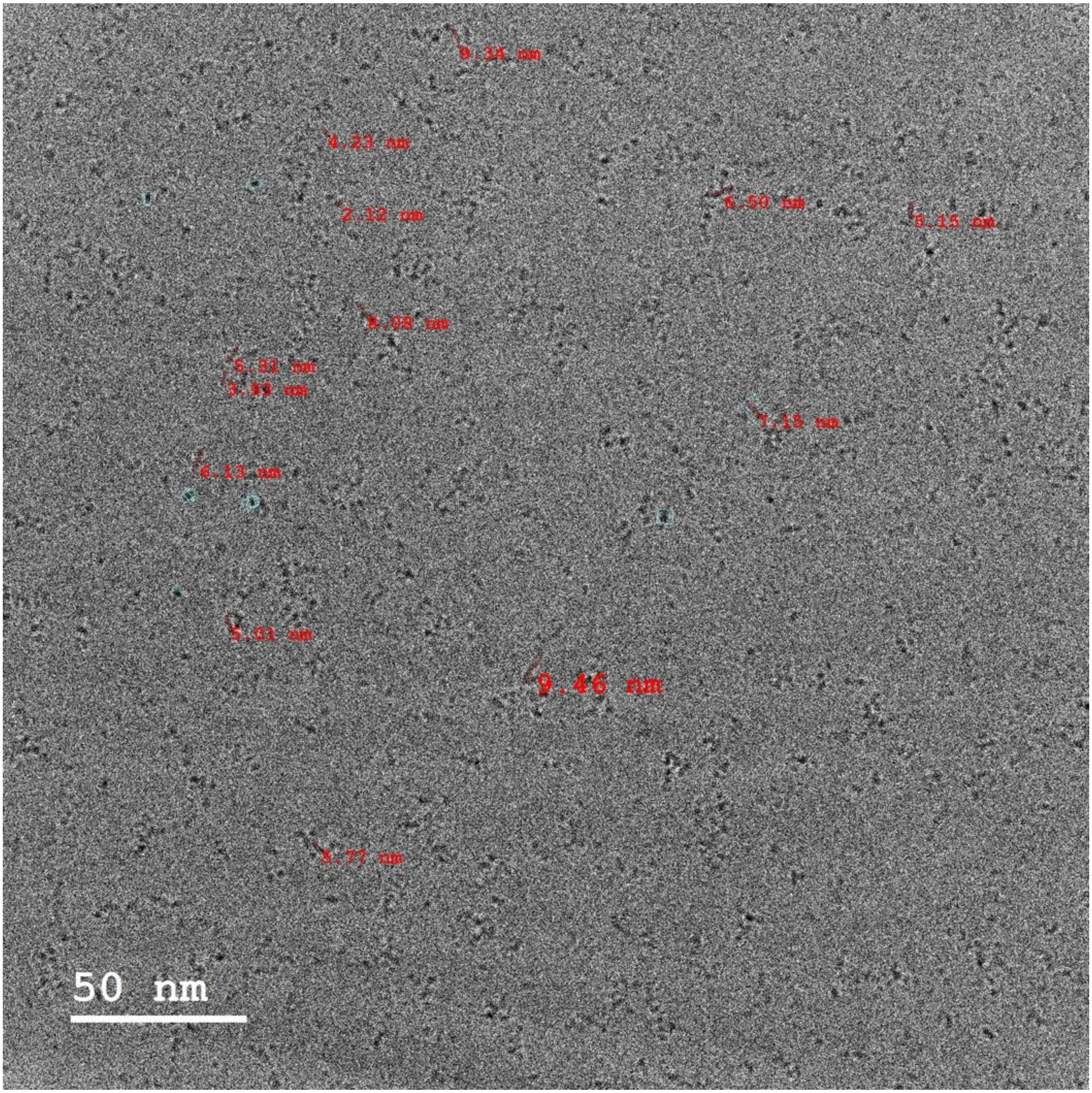

Table S1: Molecular cross sections of the aggregated IR 820

cross sections	measured value	avraged points	measured value	avraged points	fiber width	measured value	avraged points
1	2.172	29	1.561	18	1	1.671	83
2	1.997	21	1.572	21	2	1.524	50
3	1.874	23	1.638	50	3	1.746	67
4	1.864	15	1.502	15	4	1.667	43
5	1.860	19	1.562	25	5	1.805	39
6	2.017	19	1.427	18	6	1.586	49
7	2.386	22	1.503	10	7	1.599	36
8	2.094	17	1.576	17	8	1.960	64
9	2.043	25	1.692	22	9	1.520	36
10	1.966	22	1.592	27	10	1.753	61
11	2.046	21	1.515	27	11	1.598	41
12	2.118	24	1.819	24	12	1.672	31
13	1.843	23	1.516	23	13	1.520	27
14	2.139	23	1.810	27	14	1.896	25
15	2.129	19	1.529	28	15		
16	2.121	20	1.592	28	16		
17	1.976	20	1.664	26	17		
18	1.979	20	1.608	26	18		
19	2.499	20	1.596	33	19		
20	2.114	32	1.819	30	20		
21	2.304	20	1.437	28	21		
22	2.030	22	1.655	27	22		
23	1.893	20	1.518	25	23		
24	2.278	26	1.823	29	24		
25	2.071	20	1.511	27	25		
26	2.268	20	1.662	23	26		
27	1.965	20	1.374	24	27		
28	2.114	23	1.736	28	28		
29	1.828	17	1.530	54	29		
30	2.352	22	1.670	25	30		
31	1.830	18	1.511	42	31		
32	1.891	20	1.513	23	32		
33	1.895	18	1.519	26	33		
34	2.113	19	1.434	32	34		
average	2.06	719	1.59	908		1.68	652
STD	0.17		0.12			0.14	

Figure S4: DLS size distribution of IR 820 in water

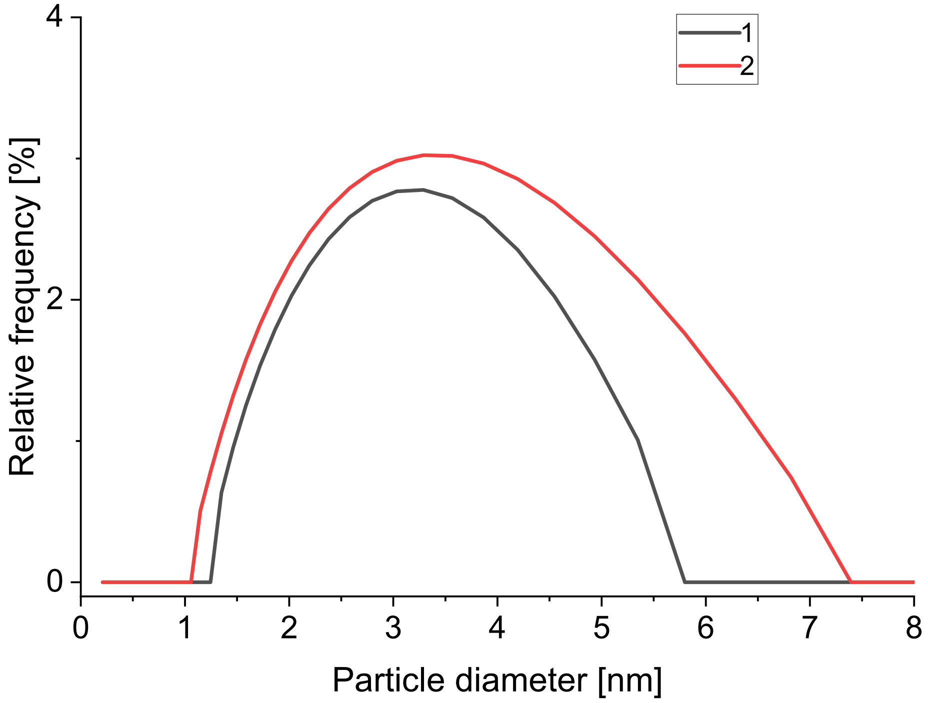

Figure S5: Fluorescence spectra of IR 820 with 800< nm short pass filter

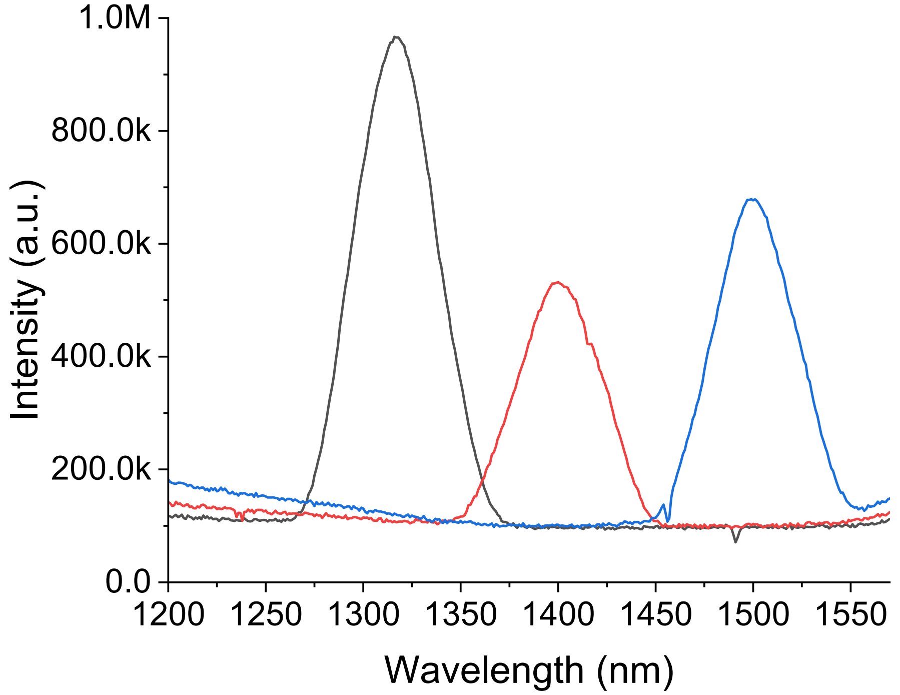

Figure S6: ^{1}H NMR spectra of IR 820 in ^{6}D DMSO

^{1}H NMR (400 MHz, DMSO) δ 8.41 – 8.34 (m, 1H), 8.33 – 8.27 (m, 1H), 8.11 – 8.04 (m, 2H), 7.86 – 7.78 (m, 1H), 7.70 – 7.61 (m, 1H), 7.57 – 7.47 (m, 1H), 6.48 – 6.37 (m, 1H), 4.42 – 4.28 (m, 2H), 2.84 – 2.72 (m, 1H), 2.04 – 1.71 (m, 12H).

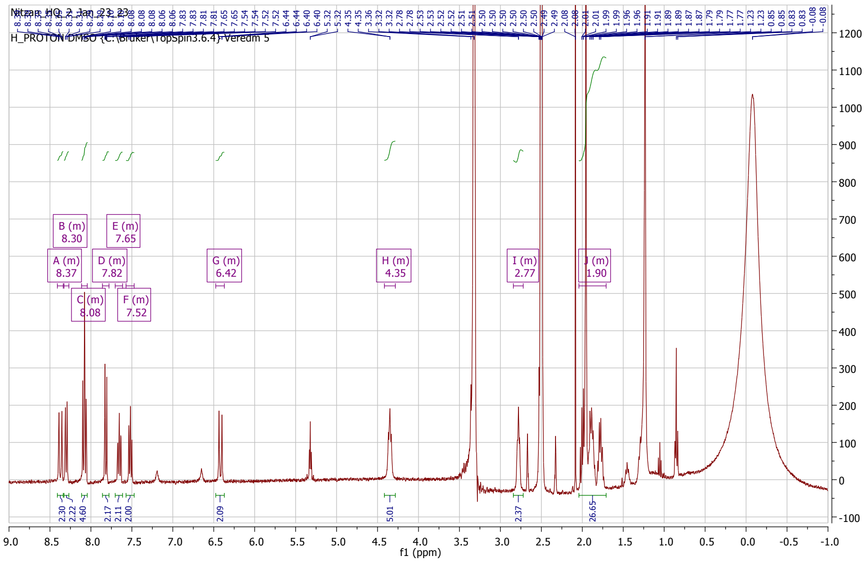

Supplement: Supplementary file 1 — jp3c04554_si_001.pdf [file jp3c04554_si_001.pdf]
